# Spatial multi-omics: novel tools to study the complexity of cardiovascular diseases

**DOI:** 10.1186/s13073-024-01282-y

**Published:** 2024-01-18

**Authors:** Paul Kiessling, Christoph Kuppe

**Affiliations:** Department of Nephrology, Rheumatology, and Clinical Immunology, University Hospital RWTH Aachen, Aachen, Germany

**Keywords:** Spatial multi-omics, Spatial transcriptomics, In situ sequencing, Multiplex in situ FISH, MALDI, Spatial proteomics, Data integration, Spatial neighborhood analysis

## Abstract

Spatial multi-omic studies have emerged as a promising approach to comprehensively analyze cells in tissues, enabling the joint analysis of multiple data modalities like transcriptome, epigenome, proteome, and metabolome in parallel or even the same tissue section. This review focuses on the recent advancements in spatial multi-omics technologies, including novel data modalities and computational approaches. We discuss the advancements in low-resolution and high-resolution spatial multi-omics methods which can resolve up to 10,000 of individual molecules at subcellular level. By applying and integrating these techniques, researchers have recently gained valuable insights into the molecular circuits and mechanisms which govern cell biology along the cardiovascular disease spectrum. We provide an overview of current data analysis approaches, with a focus on data integration of multi-omic datasets, highlighting strengths and weaknesses of various computational pipelines. These tools play a crucial role in analyzing and interpreting spatial multi-omics datasets, facilitating the discovery of new findings, and enhancing translational cardiovascular research. Despite nontrivial challenges, such as the need for standardization of experimental setups, data analysis, and improved computational tools, the application of spatial multi-omics holds tremendous potential in revolutionizing our understanding of human disease processes and the identification of novel biomarkers and therapeutic targets. Exciting opportunities lie ahead for the spatial multi-omics field and will likely contribute to the advancement of personalized medicine for cardiovascular diseases.

## Background

Organs are built from billions of cells and multiple cell types. Organ function is dependent on tight control of intrinsic and extrinsic stimuli within the tissue microenvironment to control cell fate decisions in development, health, and disease. The earliest morphometric events of an embryo occur at the 8–16 cell stage when compaction occurs [1]. Further, cell divisions, activation, and inhibition of regulators like transcription factors lead to unique tissue patterns and organ shapes, which support multiple unique organ functions. Cell-cell communication is crucial for maintaining the spatial organization of tissues and organs, ensuring the continuity of their functions across various distances [2]. In the human heart, the proper spatial organization of cells guarantees efficient energy conversion leading to synchronous cardiac muscle contractions, the rhythm of life.

For many years, anatomists and physiologists have focused their studies on cell and tissue morphology using approaches like histological staining techniques and electron microscopy. They identified various spatial features of the heart at macroscopic and at the subcellular level (e.g., distinct cell-cell contacts of cardiomyocytes, intercalated discs (ICD) [3]). While cardiomyocytes make up most of the heart by volume, they are outnumbered by a diverse mix of fibroblasts, immune cells, endothelial cells, and mural cells, which form the organ scaffold and vascular compartment [4]. This organization is disturbed in disease, often in a similar fashion across cardiovascular diseases, e.g., in the context of fibrosis or tissue inflammation. Examples include the zonation of the myocardium into distinct spatial domains of injury after myocardial infarction (ischemic zone, border zone, and remote zone [4]), vascular calcification initiated at different locations in blood vessels [5, 6] and focal segmental glomerulosclerosis (FSGS), and lesions with distinct spatial organization in fibrotic glomeruli of the kidney caused by hypertension [7–9]. Thus, methods are needed to shed unbiased insights into the spatial molecular changes of these localized processes.

Nature methods selected spatial transcriptomics (ST) as “method of the year 2020” [10], and since then, several studies applied these technologies in cardiovascular research. Single-cell and spatial multi-omics studies of the heart and kidney exemplify the recent development and insights by applying these technologies. While single-cell and single-nuclei studies of the human heart in health [11, 12] and disease [4, 13–15] have led to valuable insights, spatial information was lacking. Spatial biology enables researchers to observe and decode these complex patterns and communications of cells within their native tissue environments. Intrinsically, cells are regulated by a complex interplay of molecular regulators on several levels, which can be measured with high-throughput “omics” technologies. Multiple molecular levels can be measured currently with massive throughputs, including the genome, transcriptome, proteome, and metabolome. In the last few years, several assays have been developed to decode these layers on the single-cell level (e.g., single-cell proteomics, single-cell RNA, or ATAC sequencing). As these technologies became more broadly available to researchers worldwide, they have led to tremendous biological insights into cardiovascular diseases, including atherosclerosis [16, 17], vascular calcification [18], kidney [19–21], and heart disease [11–13, 15, 22, 23] and transformed our understanding of cellular heterogeneity, differentiation trajectories, and plasticity. The impact of these technologies is highlighted by the initiation of consortium-based research projects like the Human Cell Atlas (HCA) [24, 25] or the Human BioMolecular Atlas Program (HuBMAP) [26], which primarily focus on creating a comprehensive cellular map of the human body detailing the location, function, and characteristics of each cell type in the different tissues. However, it has become clear that for a comprehensive understanding of intrinsic and extrinsic factors which control cell fate decisions in tissues, it is crucial to consider and include spatial molecular information. Similarly to the speed of development of single-cell assays (from 1 cell [27] to 100,000 s with ultrahigh throughput [28, 29] within 10 years), the development of spatial technologies has recently gained pace, and various technological breakthroughs have led to the development of innovative approaches to study spatial biology (additionally reviewed here [30–34]). This increase in scope has made it possible to assemble spatial multi-omics experiments, opening up new perspectives on cell biology. One of the most pivotal questions is which spatial technologies, or combination thereof, to utilize for a given biological question. Here we give a comprehensive overview of the technological principles of various spatial technologies, their strengths and weaknesses, current and emerging computational strategies to analyze spatial data, challenges, and potential future directions of spatial multi-omics in cardiovascular disease.

## Spatial multi-omics technologies at cellular resolution

### NGS-sequencing-based spatial multi-omics

**Fig. 1:**
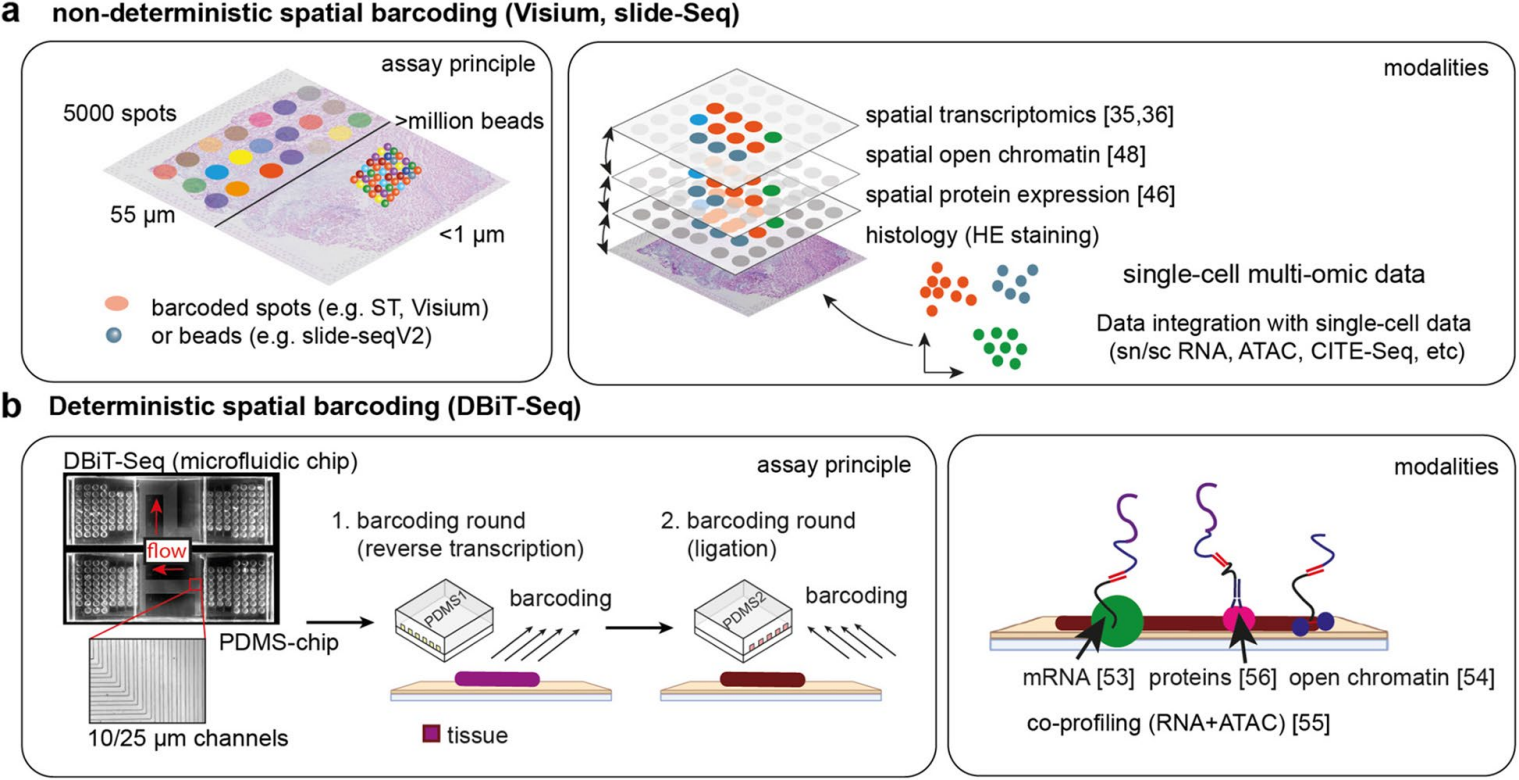
NGS-based spatial multi-omics. **a** Schematic of nondeterministic barcoding for spatial transcriptomics. Either barcoded spots (e.g., Visium) or beads (Slide-Seq) are used in an array to capture released reverse-transcribed RNA on a glass slide. Spatial multi-omic datasets can be generated using data integration with different single-cell methods. **b** Schematic of deterministic barcoding which utilizes PDMS chips with microchannels to barcode the tissue in two rounds. Several omic layers can be derived from the same tissue section including transcriptomics, proteomics, and epigenomics

The current rise of ST accelerated after the development of a high-throughput transcriptome-wide assay using arrayed oligo-nucleotide barcoded spots by Ståhl et al. in 2016 [35, 36], which formed the basis for the commercialized product called Visium by 10× Genomics. While this assay provides high throughput, the resolution is currently limited to 55-μm diameter spots arranged in a hexagonal array with a 100-μm distance between spot centers (see Table 1). Alternative array-based spatial transcriptomic methods have been developed with higher resolution and different spot barcoding principles either using beads, like Slide-Seq/Slide-SeqV2 [37, 38] or barcoded wells with 2-μm resolution-like HDST [39]. These technologies aim to close the gaps inherent to dissociated single-cell data providing information on the colocalization of cell types and cell states, spatial covariance of gene expression changes, and defining cellular tissue niches and how they change in disease (Fig. 1). Furthermore, this information is used to analyze cell-cell communication and utilized for machine-learning approaches to link the data to the patient’s clinical outcome. In the cardiovascular research space, single-cell and spatial multi-omics studies of the heart and kidney exemplify the recent development and insights by applying these technologies. While single-cell and single-nuclei studies of the human heart in health [11, 12] and disease [4, 13–15] have led to valuable insights, spatial information was lacking.

**Table 1:** Overview of spatial multi-omics methods. Experimental methods, corresponding instruments, analyte principles, modalities, feature scale, and resolution are shown for each category. The cost row offers an estimation on the price per sample and does not include initial investment into the device

	***NGS based multi-omics ***	***Imaging based multi-omics***						***MS- based multi-omics***	
	**Spatial non-deterministic barcoding**	**Spatial deterministic barcoding**	**ISS**	**Multiplex ISH – diffraction limited**	**Multiplex ISH – non diffraction limited**	**Cyclic IF**		**Ion-labelled antibodies**	**microdissection**
***Method***	Visium^35,36^, Slide-SeqV2^37,38^, STEREO-Seq^101^, HDST^39^	DBiT-Seq^53^	HybISS^92^, FISSEQ,	MERFISH^93^, seqFISH^94^, osmFISH, EEL-FISH,	FLASH-PAINT^103^, DNA-PAINT, SUM-PAINT^104^	CODEX^72^, COMET, 4i^70^, IBEX^71^, Immuno-SABER^73^		IMC^68^, MIBI^69^	Deep Visual Proteomics^77,78^ (DVP)
***Instrument***	Sequencer (short/long read)	Sequencer (short/long read)	microscope (epifluorescence confocal)	microscope (epifluorescence confocal)	microscope (confocal, TIRF, STED)	microscope		Mass-Spec	Laser-microdissection microscope + Mass-Spec
***Analyte principles***	Barcoded primer	Barcoded primer	Padlock probes	Probe panel	Oligo-labeled nanobodies	Oligo or IF labeled antibodies		Ion-labeled antibodies	Tissues markers and AI software for dissection
***Modalities***	DNA, RNA, protein	DNA, RNA, protein	DNA, RNA	DNA, RNA, protein	RNA, protein	protein		protein	protein
***Feature scale***	10.000s Protein: 100a	RNA:10.000s Protein: 100s	1000s	cells (1000s) tissues (100s)	12 (theoretically 10.000s)	1-200		1-50	unlimited
***Resolution***	1-55 µm, Stereo-seq: 200 nm	10 µm	Diffraction limited	Diffraction limited	Sub-5 nm	Diffraction limited		100 nm-1µm	5-10 µm (cellular level)
***Costs***	+++	++	++++	++++	+	+ - ++		++	++

In our spatial multi-omic study of human myocardial infarction [4], we utilized multi-omic techniques such as single-cell gene expression sequencing, chromatin accessibility sequencing (scATAC-seq), and ST to build a molecular map of cardiac remodeling, including multiple clinical time points. Our study enabled a detailed examination of unique disease markers by analyzing tissue samples collected at various intervals after MI and from distinct areas of the heart. Using this approach, we could resolve several cardiac cell types in their spatial context and the tissue microenvironment. Integrating multimodal data facilitated the identification of specific alterations in the transcriptome and epigenome in response to ischemic damage, repair, and myocardial remodeling and the establishment of gene regulatory networks.

ST approaches combined with other technologies have been used by other studies to shed light on tissue remodeling following MI in mice. Using a neonatal mouse model and ST, a recent study [40] focusing on cardiac regeneration found that the transcription factor Nrf1 regulates the oxidative stress response, which protects the neonatal heart from ischemic injury. Another study focused on the border zone (BZ) in mouse models found the gene CSRP3 to be critical for the regulation of remodeling processes after myocardial infarction and identified distinct mechano-sensing genes in the BZ of the infarct [41]. Boileau et al. introduced scNaST (single-cell nanopore spatial transcriptomics), a method targeted at the identification of RNA-isoform switches using long-read sequencing of the myocardium following infarction in different regions of the myocardium [42].

In a recent seminal study by Kanemaru et al., the researchers performed spatial multi-omic profiling of the human heart, including in a total eight regions [22]. The authors used single-cell transcriptome and multiome (RNA+ATAC) profiling to define cellular niches and to investigate cell-cell communication, primarily focusing on the cardiac conduction system. In addition, they developed a druggable target prediction tool (drug2cell) revealing the cardiac cellular targets of GLP-1 analogues. This spatial atlas will be of tremendous use for future studies involving diseased human heart tissues.

Other studies have utilized spatial transcriptomic approaches to decode the inflammatory processes in tissue space, e.g., viral myocarditis [43]. Using ST, the authors decoded the host response in neonatal mice. They observed the molecular basis of how endothelial cells mount a potent innate immune response in the heart, which is associated with localized stress response signatures. Spatial resolution was crucial for these findings, as myocarditis shows distinct zonation and border zones with unique inflammatory signatures that could potentially be missed in single-cell RNA sequencing studies of dissociated tissue. Alternative spatial transcriptomic approaches allow the user to assay selected regions of the tissue based on distinct photomasks. UV light is then used to release photoactivated linker molecules, which can be sequenced using NGS. This platform is called digital spatial profiling (DSP) or GeoMX (from NanoString). Researchers have recently used it to shed light on the human immune landscape in cardiac sarcoidosis, a lethal inflammation of the human heart [44]. The spatial measurement enabled them to find a novel marker of multinucleated giant cells and identify patterns in the location of several immune cells throughout the granuloma.

Based on transcriptomic data alone, the differentiation of naïve or mature immune cells is generally challenging, but additional protein markers such as CD45RO/CD45RA commonly used in FACS can significantly aid the identification of the correct immune cell state. Recently, spatial transcriptomics technologies have been developed to include protein information from a panel of selected antibodies similar to CITE-Seq [45] in single-cell RNA sequencing. In SPOTS [46], oligo-labeled antibodies are applied on the tissue section with a unique ID and UMI sequence, allowing for a quantitative assessment of the protein expression in addition to a measurement of the whole transcriptome. SM-Omics [47] is another ST approach which combines the capture of released RNA on an array with an oligo-label antibody staining strategy. Library generation is automated with the help of a pipetting robot, which scales up library generation to nearly 96 libraries in a little over 2 days, albeit at a resolution of only 100 μm.

Similar to spatial protein expression analysis, Llorens-Bobadilla et al. have recently adapted their ST approach to measure open chromatin information using spatial ATAC-Seq [48].

Another interesting development has been the combination of spatial transcriptomics and expansion microscopy called Ex-ST [49]. By embedding the tissue in swellable gel and expanding it up to 2.5-fold, the authors were able to reach a near single-cell resolution and also increased the capture efficiency of lowly expressed genes. A key innovation of this protocol is the use of two different poly-T oligos with different melting temperatures. In scRNA-seq, technologies, like SMART-Seq total [50] or VASA-Seq [51], extended measurements from just mRNA to all other RNA species like miRNAs, lnc-RNAs, and non-host RNA, like viral transcripts, by enzymatic polyadenylation. Analogous, a spatial total RNA sequencing (STRS) method has been recently developed [52].

While the technologies described above rely on the diffusion of reverse-transcribed transcripts onto immobilized oligo-dT-nucleotides barcodes, in DBiT-Seq [53], microfluidic channels in PDMS chips are used to actively flow the barcodes on the tissue (see Table 1). Sequential barcoding in the reverse transcription (barcode A) and ligation step (barcode B) is used to individually and deterministically barcode tissue pixels using DNA-oligos. This method has been recently adapted to measure spatial ATAC-seq profiles [54] and co-profiling of RNA and ATAC or other epigenetic features like histone modifications in the same tissue section [55] as well as proteomics [56] (Fig. 1). In 2022, this technology was highlighted with other spatial multi-omics methods as one of “seven technologies to watch in 2022” [57]. The resolution of DBiT-Seq is determined by the diameter of the PDMS-chip channels, which range from 10 to 50 μm, thus reaching near single-cell resolution. The versatility of this approach, especially in regard to multi-omics, is remarkable and could in theory be extended to other modalities like 3D genome organization [58], APEX-seq [59], or higher throughput as demonstrated recently [60].

Kishi et al. presented another innovative barcoding strategy combining in situ barcoding and ex situ NGS sequencing called Light-seq [61]. A unique combination of technologies allowed the authors to specifically target a very rare cell type, dopaminergic amacrine cells (DAC cells) of the mouse retina, which otherwise would be very difficult to capture. Another distinguishing feature, compared to all other spatial methods, is that it leaves the original sample intact, opening several opportunities for further downstream analysis using other omics. Furthermore, this technology might be extended to analyze the proteome or epigenome.

An exciting development for single-cell [62–64] and spatial transcriptomics [65] has been the recent adaptation to formalin-fixed paraffin-embedded (FFPE) tissues. Several challenges had to be overcome, including the development of FPPE-specific nuclei isolation protocols [64] and strategies to handle RNA cross-linking and RNA degradation typical to FPPE tissue stored at room temperature. The 10× Visium workflow, originally designed for cryo tissue, was adapted to the FFPE tissue workflow by the inclusion of three pairs of probes for each target mRNA. The assay measures mRNA transcriptome wide and can be refined by the spike-in of custom probes. Since FFPE samples are the gold standard for tissue preservation for pathologists (mostly due to their excellent ability to preserve tissue morphology) and FFPE tissue is widely accessible, the development of these approaches is particularly promising. Clinical data of these samples are usually available, thus enabling the study of larger retrospective cohorts with detailed metadata. A recent study combined the probe panel Visium FFPE workflow with low-quality fresh-frozen samples (FF), which led to great improvements in data quality and even made it possible to spatially profile cartilage and bone tissues in mice [66], signifying light at the end of the tunnel for the processing of these challenging samples.

### Non-NGS-based spatial multi-omics

While transcriptome information is widely used to model protein expression dynamics, the genome-wide correlation between mRNA and protein is estimated to be only around 40% [60, 61]. Furthermore, the transcriptome alone cannot provide information about processes induced by posttranslational protein modifications, which can have important effects on cell biology. Compared to the transcripts, resolving the proteome at single-cell resolution or in tissues is much more challenging. Cells typically contain 30,000× more protein molecules than mRNA molecules [67], and proteins are very heterogeneous in size and structure and, unlike nucleic acids, cannot be amplified. This has limited the multiplexing and throughput of spatial proteome measurements. Nevertheless, enormous progress has been made in generating multiplex proteomics datasets from tissues.

Antibody-based multiplexed imaging technologies have been available before NGS-based spatial assays relying on mass cytometry for IMC-Cytof [68] or MIBI-TOF [69] but have only recently increased in throughput (larger field of view > 1 cm^2^) and complexity (> 10–50 markers) (see Table 1). The development of fluorescence-based technologies such as 4i [70] and IBEX [71] and DNA-oligo labels in the case of CODEX [72] and Immuno-SABER [73] has made spatial proteomics more approachable, as researchers do not need access to MS instruments.

A major challenge in antibody-based proteomics is validating the antibody specificity and ensuring that it is not influenced after the conjugation step with fluorophores or DNA. Another approach to enable unbiased single-cell or spatial proteomics is based on highly sensitive LC-MS-based proteomics (reviewed here [67, 74–76]), which has been adapted to spatial proteomics as deep visual proteomics (DVP) [77, 78]. DVP combines laser-capture microdissection of distinct cell types from tissue and performs MS proteomics of the collected tissues extending bulk proteomics of the human heart [79] with cell type and spatially resolved information in the near future.

Recent developments of transgenic mice have enabled metabolic labeling of proteins using Cre-recombinase-induced expression of a mutant methionyl-tRNA synthetase [80, 81] from a given cell population. Combined with unbiased MS-proteomic approaches, this might be well suited to resolve the cell secretome or for the discovery of novel biomarkers, which otherwise might be missed in proteomics of isolated cells.

Spatial metabolomics has reached 5–10 μm resolution and has been recently applied to study cell-type-specific dynamics of metabolism in kidney repair [82]. Every year, 13 million people suffer from AKI and increased cardiovascular risk burden [83]. The kidney tubules can regenerate following AKI; in most patients, the injury resolves via adaptive regeneration [84]. How this process is molecularly wired is unknown, yet the importance of metabolic factors contributing to this process is widely recognized. MALDI-MSI-based metabolomics combined with 13C-labeled nutrients allowed the authors to study the dynamics of metabolic changes at subcellular resolution. They subsequently used multiplexed immunofluorescence microscopy to identify cell types which seemed unnecessary, as cell types could be differentiated just based on the lipid profiles.

One area where spatial metabolomics might offer great value is research targeting the protective effect of SGLT2 inhibition in renal proximal tubular cells. Inhibition of SGLT2 in the proximal tubule demonstrated a remarkably beneficial effect on survival in patients suffering from cardiovascular disease, including heart and kidney disease [85]. Secondary molecular changes are however not well understood. Spatial metabolomics can elucidate this potent pathway and potentially lead to novel targets.

Additional developments and progress in spatial metabolomics have been recently reviewed here [86, 87]. The correct metabolite annotation of MS data remains a particular challenge. Resources like www.metaspace2020.eu offer a powerful platform for annotating and sharing metabolic MS datasets.

A combination of spatial multiplexed IF imaging and spatial metabolomics has recently been established to investigate myeloid cell heterogeneity in atherosclerotic plaques [88], shedding light on plaque myeloid phenotypes.

One recent approach demonstrated the measurement of metabolites or other arbitrary targets based on NGS structure-switching aptamers [89], which are constructed to release barcodes upon contact with the target molecules in single-cell RNA sequencing assays. Follow-up studies on this interesting strategy must be performed to determine how scalable these approaches are. While certainly not on the immediate horizon, single-molecule protein sequencing might be combined with MS and antibody-based multiplex imaging approaches to shed light on the proteome at unprecedented resolution. A recent review [90] is included here for completion.

## Spatial multi-omics technologies at single-molecular resolution

Several approaches have been developed which allow in situ sequencing (ISS) or imaging-based fluorescence in situ hybridization (FISH) of RNA, DNA, or proteins. In ISS, mRNA is labeled with specific nucleotide sequences called padlock probes [91], which are then sequenced with rolling circle amplification to increase the specificity of fluorophore binding (see Table 1). HybISS [92], or the commercialized version by 10× Genomics (Xenium), enables the detection of 100–1000 targets at subcellular resolution, including the detection of mutations. In general, these approaches have a very high sensitivity allowing them to detect lowly expressed genes.

FISH-based technologies like MERFISH [93] or SeqFISH+ [94] encoding probes are designed based on a binary barcoding scheme, which allows for error correction during the readout of the fluorescent barcode (see Table 1). These methods have been recently developed to measure open chromatin [94] and simultaneously 3D genome, proteome, and transcriptome [95]. The measurement of high-resolution transcriptomics using MERFISH in > 50–100 μm section has recently been proposed [96].

In general, both ISS- and FISH-based methods require the selection of a limited number of targets to form a probe panel *a priori*. Computational approaches like spapros [97] and others [98, 99] provide a workflow to identify these genes based on reference single-cell RNA sequencing data.

Several other technologies are available: CosMx [100] (NanoString), and Molecular Cartography (Resolve Biosciences). However, an independent benchmark that compares sensitivity, specificity, and other performance metrics has not been carried out.

Unbiased ST methods at subcellular resolution have been developed recently, such as Stereo-Seq [101] and SeqScope [101]. While these approaches allow subcellular resolution transcriptomics (e.g., 220-nm diameter size for Stereo-Seq), assignment of signals to specific cells is potentially more challenging, as cell boundary stains are not available and diffusion dynamics might affect the measurement. It remains to be seen how accurately these spatial assays perform compared to other in situ approaches.

These technologies enable additional insights into subcellular spatial localization of molecules, e.g., between the nucleus or cytoplasm, to gain insights on transcriptional dynamics of cell states. They have not been extensively applied in cardiovascular research since they are quite novel and just starting to be accessible to more researchers worldwide.

While these technologies have already reached an astonishing resolution, recent developments of DNA-PAINT approaches like FLASH-PAINT [102] or SUM-PAINT [103] and the development of Ångström-resolution fluorescence microscopy (RESI) [104] might lead to even further increases in multiplexed detection of biomolecules at nanometer ranges. Particularly, the combination of these DNA-imager-based approaches with DNA-based protein-binding aptamers [105] (SOMAmers) might open the possibility to profile 1000 s of proteins in situ below the diffraction limit at single protein resolution in the future.

## Computational approaches for spatial multi-omics

New experimental designs also require innovative approaches for data analysis. Adding the spatial dimension to multi-omic data sets poses significant challenges but empowers existing analysis tools and opens entirely new ways of understanding tissue biology. In this section, we will discuss data analysis strategies and key challenges of working with multi-omic spatial datasets. For a more in-depth technical perspective, readers are directed to additional recent reviews on this topic [106–108] and the community resources at www.sc-best-practices.org and https://lmweber.org/BestPracticesST.

Computational workflows are dependent on the technology that produced the data, but there is significant overlap in the processing of the different modalities. The objective of these workflows is similar: to link the signal recorded by the instrument, be that a fluorescent intensity or barcode, the sequence of reverse-transcribed RNA, or the m/z value of an ion back to a spatial location in the tissue. Technologies with cellular resolution are then able to assign signals to individual cells in a process known as cell segmentation.

### Cell segmentation

**Fig. 2:**
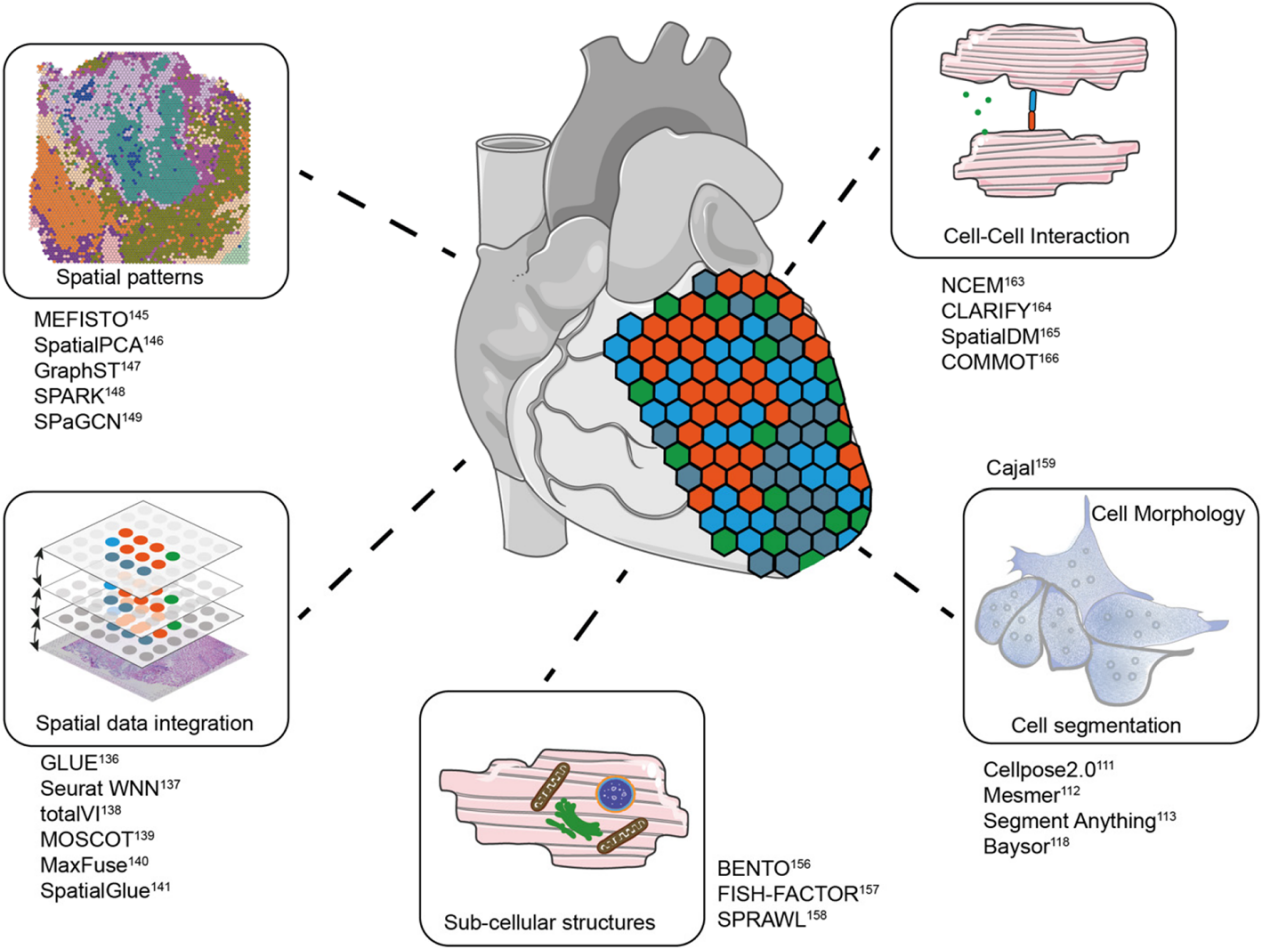
Overview of key areas of analysis enabled by spatial multi-omics and example software implementations. Gene expression in tissues is not random but forms characteristic spatial patterns. Preserving the spatial context allows for the analysis of cell-cell interaction at different length scales. Imaging-based technologies enable the extraction of further features like cell morphology or sub-cellular structures but require well-optimized cell segmentation. A key area of software development is spatial data integration

Cellular segmentation of tissues is often improved by combining the primary readout with additional stains, such as DAPI staining of nuclei and or the cell borders with anti-cadherin antibodies or similar compounds. In imaging-based approaches like FISH, ISS, or multiplexed immunofluorescence, these measurements can be acquired simultaneously with the main measurement, but even measurements that do not require a microscope like NGS-based Stereo-seq [109] or MALDI measurements [110] are often co-registered with separately acquired microscopic images. To assign the measured signal to biological entities like cells or nuclei, the positions of the entities need to be extracted from the image in a process known as instance segmentation of cells. This step is critical, as misassignment of the signal can contaminate the measurement with cells which present a mix of signals originating from different cell types or can entirely hide difficult-to-segment entitles from downstream processing. Deep-learning-based segmentation algorithms like Cellpose 2.0 [111], Mesmer [112], and Segment Anything [113] have shown superior performance to more traditional algorithms like watershed [114] but are highly sensitive to cell diameter and shape. This is problematic, as tissues like the heart are composed of cells with vastly different morphology. Human cardiomyocytes are cylindrically shaped with an approximate length and diameter of around 100 μm and 20 μm, respectively [115], which is much larger than interstitial or immune cells for example. Furthermore, they can be multinucleated [116] and vary in shape based on the sectioning of the tissue and disease progression [117]. This poses significant difficulties for deep-learning algorithms, which were not trained on a large corpus of heart data, necessitating fine-tuning. A promising direction for cell segmentation is the probabilistic assignment of signals with tools like Baysor [118], ClusterMap [119], or Sparcle [120], which employ statistical models which judge the likelihood of transcripts originating from the same cell. In technologies where cellular resolution is impossible, the signal is instead assigned to regions of interest or binned into tiles representing the resolution limit (Fig. 2).

The result of this processing is an entity-by-signal-by-position matrix compatible with a variety of downstream analysis workflows. Ecosystems that process spatial multi-omics data in a standardized way are just beginning to emerge. Of note are SpatialData [121], SpatialExperiment [122], GiottoData [123], and SeuratObject [124], which offer data containers that can store the diverse data generated by spatial-omics experiments. This infrastructure is critical to unify the heterogeneous data from different technology-specific vendor formats and the various images, tables, and polygons that a single experiment can generate and makes the development of analysis algorithms that “plug-and-play” possible.

### Integration of multi-omic datasets

To unlock the synergy of multi-omic measurements, integrating the datasets with each other is necessary. This is a nontrivial task as the data generated by different modalities exist in entirely different feature spaces. When talking about integration, it is important to differentiate several scenarios. Argelaguet et al. [125] proposed three categories of integration tasks: *Horizontal* integration is used when the same modality is measured across different samples. An example could be the measurement of the spatial transcriptome across multiple myocardial infarction samples. *Vertical* integration describes the integration of measurements in the same cells, such as co-profiling of the epigenome and transcriptome in DBiT-seq. The most challenging case is *diagonal* integration. Here, neither the cells nor features are shared across datasets, and integration instead relies on a strong biological signal that can be captured with different modalities independently from each other. When applying MALDI and spatial transcriptomics to alternating sections of the heart, neither the features nor, depending on the thickness of the slice, the cells are shared between experiments.

A special case, often referred to as imputation, is the enrichment of targeted spatial transcriptomics with whole transcriptome sc-RNAseq reference data. These measurements, which are often restricted to 500 or fewer species of RNA, can thus be virtually extended to cover the whole transcriptome. This task is comparatively easier, as the concordance between spatial RNA measurements and single-cell RNA seems to mostly hold across genes [126, 127]. Notable software packages in this domain are Tangram [128], gimVI [129], and ENVI [130].

One approach for horizontal integration is the generation of a common coordinate framework, a shared coordinate system across measurements [131–133]. Based on common landmarks, and distinct morphological features of tissues, slices are morphed and aligned to overlap in space. With enough measurements, this system can then be used to construct entire 3D organs [134, 135].

In vertical and diagonal integration tasks, the collected data needs to be projected into a common latent space to minimize various distance metrics. In general, algorithms developed for single-cell experiments like GLUE [136], Seurat WNN [137], or totalVI [138] could be applied to spatial experiments; however, this would dissociate cells from their spatial context and ignores the cellular neighborhood, which is highly informative for integration tasks. A new class of tools developed specifically for the integration of spatial experiments has recently come into focus. Moscot [139] formulates integration as an optimal transport problem, encoding the Euclidean distance among spatial locations, MaxFuse [140] applies graph-smoothing to the input data and iteratively matches modalities after co-embedding, while SpatialGlue [141] employs a graph neural network (GNN) and uses an attention aggregation layer to integrate constructed spatial proximity and feature graphs (Fig. 2).

While these tools show promise, it is important to note that as of the time of this article’s publication, several tools have not undergone peer review. An independent benchmark of spatial-omics integration algorithms is urgently needed and would help scientists in their choice of integration strategy and inform their experimental designs.

A well-integrated dataset is very powerful for a lot of common downstream analysis tasks. Cell typing is much improved when information about both transcriptome and proteome is available, as cells like NK-cells, which play a major role in inflammatory heart disease [142, 143], are notoriously hard to classify based solely on their low transcript counts. The combination of epigenome and transcriptome reveals clear links between gene expression and transcription factor binding and enables the construction of gene regulatory networks, e.g. the recently develop SCENIC+ tool [144], across spatial domains like the border zone of myocardial infarction [4] or pacemaker cells [22].

In heart disease, inflammatory and fibrotic responses are not randomly located in the tissue but possess specific motifs or principles. In MI, *ANKRD1* and *NPPB* show a gradient across the border zone of the infarct [4], and the epicardium has been shown to contain distinct niches of plasma B cells [22].

A variety of tools have been tailored to facilitate similar discoveries. Analogous to the conventional processing of single-cell experiments, it is possible to create lower dimensional representations of spatial data to identify trends across cells. MEFISTO [145], SpatialPCA [146], and GraphST [147] allow for the identification of clusters of cells not only based on the measured signal but also based on the tissue niche and the surrounding types of cells, leading to a more fine-grained understanding of tissue structure principles. Another branch of tools like SPARK [148], SpaGCN [149], or SpatialDE [149, 150] investigates patterns in feature expression across the region of interest, often with additional functionality like pseudo-time analysis in the case of SpaceFlow [149–151] or the detection of patterns across consecutive slices in STAGATE [152] (Fig. 2).

With the introduction of spatial omics with subcellular resolution, this search for patterns is not limited to a macroscopic view of tissue composition but can also be extended to structures inside cells. The cellular location of proteins is often disturbed in disease like in familial atrial fibrillation, for example, which is caused by a mutation that impedes HSP70 import [153]. The role of RNA localization remains poorly understood but has been implicated in developmental processes [154] and diseases like Huntington’s disease [155]. Recently published tools like Bento [156], FishFactor [157], and SPRAWL [158] analyze the position of RNA inside cells and try to identify subcellular compartments and principles of transcripts across the cytoplasm and nucleus (Fig. 2).

Cell morphology is another resource that remains underutilized in the analysis of spatial datasets. Cardiomyocytes and fibroblasts are known to change their cellular phenotype and morphology in response to stress, such as hypertrophy, elongation, or thickening [117]. These features offer valuable insight into cell state that could be integrated with existing omics measurements. Cajal is an algorithm that transfers cell shapes into a latent space that can be integrated with genomic readout [159].

### Cell-cell interaction in space

Cells in the heart are engaged in intense cross talk with their cellular niche. G-protein-coupled receptors, ion channels, and their paracrine and autocrine signaling are only some examples of critical communication circuits in the development of heart disease [160–162]. These processes can be studied on the transcriptional level based on the expression of key receptor and ligand proteins and curated databases that collect matched receptors and ligands. Until recently, programs had to rely on dissociated single-cell data for this task. This is however problematic, as most forms of cell-cell communication are short ranged and very much dependent on the spatial tissue organization.

A new generation of algorithms combines receptor-ligand analysis with the position of molecules to investigate these key processes (Fig. 2). NCEM [163] and CLARIFY [164] are neural networks which model gene expression as a function of their spatial neighborhood, SpatialDM [165] applies bivariate Moran’s statistics, and COMMOT [166] is based on collective optimal transport. It remains to be seen which approach yields the most biologically relevant information, as no comparative benchmarking has been published so far.

## Design considerations for spatial multi-omic experiments

Before starting a spatial multi-omic experiment, several key questions have to be addressed by the researcher to inform the choice of technology and the design of the experiment. On the one hand, these are imposed by the research question. The choice of which modalities to measure and at what resolution cannot be answered by a general guide but must be made on a project-to-project basis. As an example, rare cells, like neuronal cells or pericytes, would best be studied with a high-resolution technique, as detection might not be possible in lower resolution measurements which conflate multiple cells into spots. The analysis of lowly expressed genes like transcription factors is best served by approaches with maximal sensitivity, such as ISS or ISH.

Experiments can in general be divided into exploratory investigations that aim to generate hypotheses and confirmatory experiments that aim to prove a well-designed research question. Generally speaking, methods with a larger feature scale such as NGS-based multi-omics and microdissection MS lend themselves well to an exploratory setting, while more targeted approaches like imaging-based multi-omics and ion-label MS shine in confirming well-defined research questions.

On the other hand, the current technical limitations of the technologies and budgetary considerations must also inform the choice of method. Spatial multi-omics experiments are costly and complex to establish and should only be employed in settings where a clear zonation of the tissue is expected. Almost all technologies require the procurement of additional devices and access to specialized facilities like NGS and mass spectrometry core facilities. Community established technologies such as DBiT-seq and HybISS are generally more affordable than their commercial counterparts, which offer convenience and a support structure at a surcharge. Comparing the initial investment required, spatial deterministic barcoding ranks at the lower end, as a house vacuum and PDMS chip are easily accessible. Spatial nondeterministic barcoding, ISS, diffraction-limited multiplex ISH, and cyclic IF fall in the medium price range, while multiplex ISH beyond the diffraction limit and MS-based multi-omics fall into the upper price segment, driven by the high cost of super resolution microscopy and a mass spectrometry + laser-microdissection setup, respectively. The upkeep costs of the devices and the cost per area of tissue (shown in Table 1) warrant further consideration.

The measurement area varies wildly between technologies from a range of mm^2^ up to 13.2 cm × 13.2 cm in the case of Stereo-seq. This not only limits what kind of samples can be measured but also has a direct effect on analysis. It has been shown, for example, that tumor samples which contain a large number of heterogeneous cell states require a high number of FOV for adequate analysis [167].

Not all technologies are compatible with all samples. While they are more widely available, samples preserved in FFPE can be more challenging to assay as these samples tend to suffer from increased RNA fragmentation and increased modifications of proteins and metabolites. A further consideration is the species of interest. Spatial multi-omic methods can in principle be applied to any kind tissue, but hybridization-based technologies such as Visium v2 or Xenium rely on the construction of probes, which are only available for human and mouse samples currently.

After a technology has been chosen, it is generally necessary to optimize the processing of samples. Different tissues can be more or less challenging and require different treatment, owing to factors such as ease of permeabilization, resistance to proteinase, or increased autofluorescence. These pilot experiments can then also be used to inform the overall design of a study. *In silico* tissue generation tools are able to probabilistically create spatial multi-omic data based on prior knowledge [167–169]. These artificial datasets can then be used in a power analysis to determine necessary FOV sizes, number of views, and replicates to answer the research question.

## Challenges of spatial multi-omics

### Experimental challenges

Many experimental challenges exist for spatial multi-omics. As with single-cell RNA sequencing, standardized sample preparation is key for successful experiments and high data quality. Especially for the handling of tissues, standardized protocols need to be followed for tissue sampling, fixation, freezing, and tissue sectioning. Protocols such as FixNCut [170] are based on Lomant’s reagent/DSP or treatments with VivoFix [171] reversible fixate tissues before dissociation, which limits artifacts induced by temperature and enzymatic digestions and preserves RNA integrity. A guide on how different tissues should be handled for each spatial multi-omics technology is currently lacking but would greatly increase reproducibility in the field.

For antibody-based multiplexed imaging methods, the specificity of a given antibody panel is a potential concern. While established and validated antibody panels are available from commercial vendors (e.g., from Akoya Biosciences for CODEX), they are associated with increased costs. In cases where custom antibodies or antibody combinations are necessary, their validation can be time-consuming, and antibody specificity can often remain unclear. Community efforts like the recent establishment of organ mapping antibody panels, OMAP [172], are needed to guide antibody selection and ensure high reproducibility.

An open question in the field of array-based spatial transcriptomics is the effect of diffusion artifacts. Especially, high-resolution array-based approaches like Stereo-Seq with 220-nm pixel resolution [109, 173] or Seq-Scope [101] might be affected by diffusion artifacts, and it is unclear if this problem might apply for future developments of array-based ST approaches with higher resolution (like Visium-HD). Aided mobilization of the barcoded nucleotides using electrophoresis, like in EEL-FISH [174], might mitigate this problem.

Furthermore, it is unclear how to best select the necessary samples and sample numbers and/or size of the field of view (FOV) for a cohort or study to accurately capture the targeted biological processes. Several *in silico* tissue spatial multi-omics generation pipelines have been established [168, 175] to explore this question. However, this might still not lead to the desired result if the tissue does not include the targeted process in the first place. Another approach could be to use machine learning methods like pathomics [176] on large-scale digital histological data, which highlights areas of interest for a given group of diseases and maximizes the amount of information which can be derived from spatial experiments.

### Computational challenges

Spatial multi-omics data analysis presents various challenges that the field will need to eventually overcome. Cell segmentation remains challenging for all imaging-based approaches, as discussed above. It is often unclear which algorithm produces the best result, as the generation of ground truth for performance evaluation relies on tedious manual annotation of the target tissue. Furthermore, the performance of segmentation algorithms is highly tissue and disease-state dependent. A pipeline that ranks the performance of several tools compared to prior knowledge of the tissue of interest might help alleviate this key problem.

Data integration is another key area where improvements need to be made. At the moment, the parallel measurement of multiple modalities of the same cells in a “true” multi-omic experiment is very limited, and researchers instead rely on algorithms that diagonally integrate separate measurements or impute missing data. This emphasizes the importance of reliable integration. It is necessary to scrutinize integrated datasets for biological plausibility, especially as a recent review of transcriptomics integration methods found that no tool reached a Pearson correlation coefficient of more than 0.5 compared to ground truth [177]. Multi-omic integration is likely even more challenging as the link between datasets is weaker. One possible way forward might be the creation of foundation models for spatial multi-omics, analogues to efforts in natural language processing [178], and single-cell transcriptomics [179–181]. These models, trained on a large corpus of spatial and single-cell multi-omic datasets, might be able to deconvolute underlying patterns necessary for successful integrations.

Lastly, spatial multi-omics experiments are challenging just based on the amount of data generated. An imaging-based spatial transcriptomics measurement can generate around 5 TB of raw data (e.g., MERFISH, own experience), necessitating a robust data storage strategy to which many academic laboratories might not have access. Research is collaborative, and sharing generated data with publications is a key pillar of reproducible science. In the case of spatial multi-omics, the large data volumes and the diversity of generated data between high-resolution images, sequencing data, and tabular information hinder this. Multiple platforms have been established to facilitate the sharing of data and easy in-browser viewing of datasets [182–187]. However, these repositories remain underused, with every website only containing a small subset of published data. Ideally, scientific journals would require sharing of data in a digestible and convenient fashion as offered by these data stores.

## Future perspectives

**Fig. 3:**
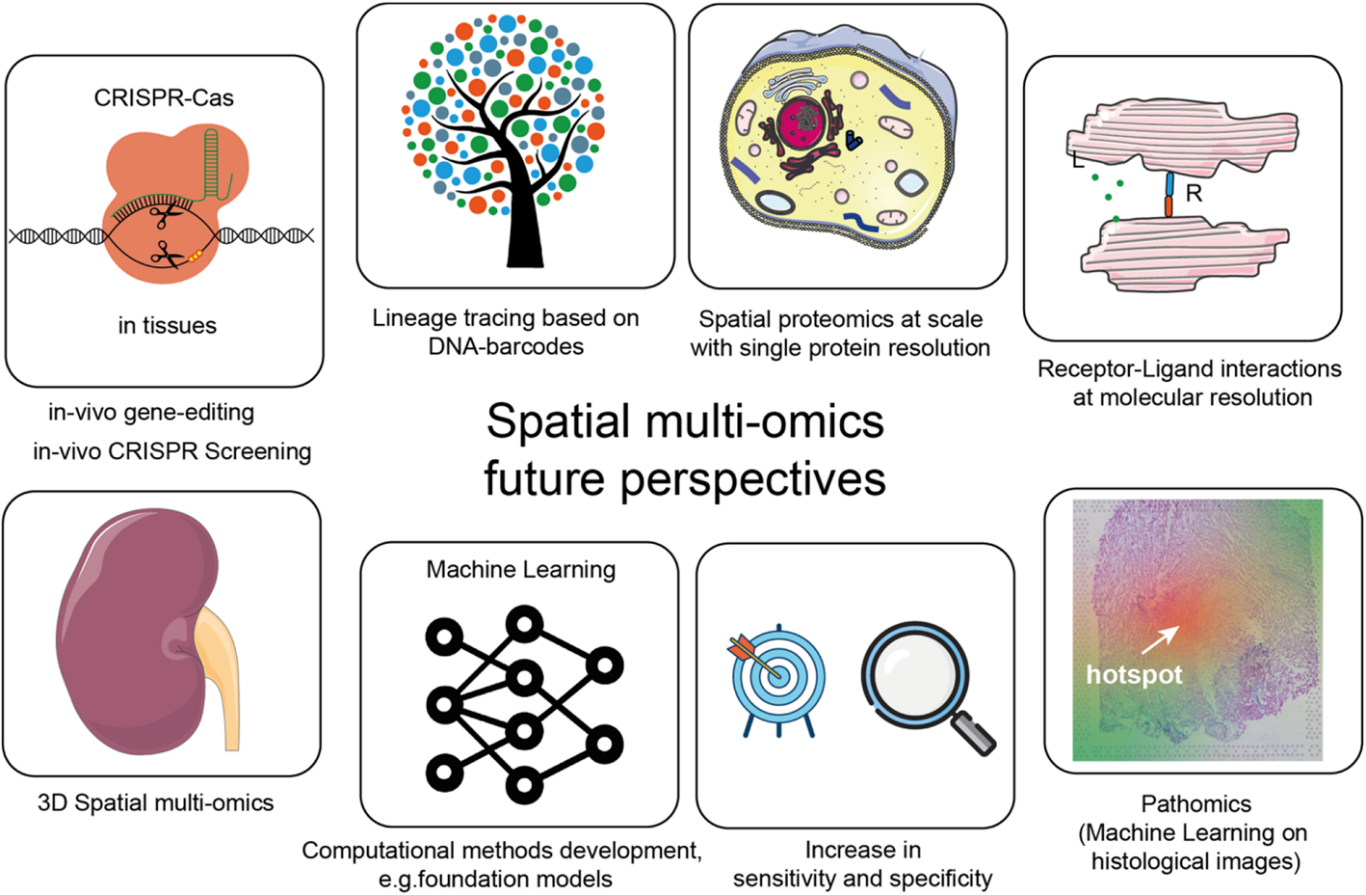
Future perspectives for spatial multi-omics. We foresee several avenous to be implemented in spatial multi-omic experiments. These include the analysis of *gene-edited tissues* using CRISPR-Cas or other editing/perturbation approaches. Additionally, gene editing can be used to *barcode cells* to enable *lineage tree reconstruction* based on spatial data. *Receptor-ligand interactions* at high resolution would greatly improve CCC analysis. *Pathomics* could guide selection of insightful tissue areas which are predictive for a disease outcome. Increased sensitivity and specificity will also certainly be addressed. Advancements in using machine learning models like foundation models will potentially help to overcome data integration difficulties. 3D spatial multi-omics data might be acquired directly or inferred from the registration of measurements in a common coordinate framework

In the future, multi-omics technology at spatial single-cell resolution will revolutionize our understanding of cell biology. Anticipated advancements include enhanced throughput, cost reductions, and integration of more modalities per assay with improvements in sensitivity and specificity. The construction of 3D tissue maps by predicting molecular features from histopathology may be a powerful approach [188]. Current challenges, such as comprehensive mutation profiling at single-cell level and co-detection of epigenomic features, will most likely be overcome soon, as demonstrated in recent publications [189]. Current proteome assays will need to evolve from antibody-based techniques to unbiased, low-input methods, as exemplified recently in the DVP workflow [77] or based on protein-binding DNA aptamers (SOMAmers, e.g., SOMALogic). Spatial assays that will allow us to decode the cell-cell interactions based on the co-detection of ligands and receptors on the protein level will be crucial to enhance the CCC modeling. Enhancing computational accuracy of data extraction from each molecular layer and integrative analyses across modalities will be crucial and will further improve predictive modeling like a *weather forecast* of biological events in tissues (e.g., acceleration of inflammation or fibrotic processes or metastasis in cancer). Additionally, one can predict a rise in the combination of gene-editing experiments and spatial multi-omics. Similar to single-cell-based CRISPR screenings, these assays can be implemented in vivo or in vitro in organoids or bioprinted constructs and spatial gene or drug perturbations consequences analyzed, thus extending the functional analysis toolbox (Fig. 3).

While several barcoding technologies exist to uncover the hierarchical structure or lineage tree of cellular differentiation on single-cell level, we expect that these technologies will be more employed in tissues like in intMEMOIR [190] to uncover hierarchical tissue maps. These might also be applied to human tissue based on somatic mutations or mutational signatures from mitochondria [191] or leveraging clonotype information from, e.g., the TCR of T cells. Furthermore, they may be combined with other digital recording systems for biological events [192, 193], further refining insights for *biological time traveling* in tissues. While these systems can be genetically introduced in vivo, one can also foresee a combination with in vitro models in which they can be more easily scaled to larger throughput. For data analysis, we envision that large-scale foundation models, which have been recently employed for the analysis of single-cell RNA sequencing data, will spur advancements in spatial multi-omics data analysis in a diverse range of downstream tasks, including data integration, cell-type annotation spatial gene expression analysis, and perturbation prediction, e.g., from drug and genetic perturbations and gene network inference. Together, these advancements in spatial multi-omics technologies and computational approaches are set to enhance our understanding of biology in health and disease and enhance the identification of markers for diagnostic and prognostic evaluation of cardiovascular diseases and novel therapeutic targets for personalized medicine (Fig. 3).

## Data Availability

Not applicable.

## Abbreviations

ICD: Intercalated disc
MI: Myocardial infarction
HCA: Human Cell Atlas
HuBMAP: Human BioMolecular Atlas Program
IHD: Ischemic heart disease
ATAC: Assay for transposase-accessible chromatin
S: Spatial transcriptomics
scNaST: Single-cell nanopore spatial transcriptomics
BZ: Border zone
PDMS: Polydimethylsiloxane
MERFISH: Multiplex error robust fluorescent in situ hybridization
MALDI: Matrix-assisted laser desorption/ionization
FFPE: Formalin-fixed paraffin embedded
FOV: Field of view
OMAPs: Organ mapping antibody panels
CODEX: Co-detection by indexing
EEL-FISH: Enhanced electric fluorescence in situ hybridization
FISH: Fluorescence in situ hybridization
NGS: Next-generation sequencing
DBiT: Deterministic barcoding in tissues
MS: Mass spectrometry
CCC: Cell-cell communication
DAPI: 4′,6-Diamindino-2-phynylindol
ISS: In situ sequencing
CITE-Seq: Cellular indexing of transcriptomes and epitopes by sequencing
FFPE: Formalin-fixed paraffin embedded
NK cells: Natural killer cells
